# Supplementary material for: Prospective Observational Study of Prevalence, Assessment and Treatment of Pancreatic Exocrine Insufficiency in Patients with Inoperable Pancreatic Malignancy (PANcreatic Cancer Dietary Assessment—PanDA)
Source: Cancers (Basel). 2023 Apr 13;15(8):2277. doi: 10.3390/cancers15082277 (PMC10136984; doi:10.3390/cancers15082277)
Supplement: Supplementary file 1 [file cancers-15-02277-s001.zip › cancers-2298521-supplementary.pdf]

Supplementary Materials: Prospective Observational Study of Prevalence, Assessment and Treatment of Pancreatic Exocrine Insufficiency in Patients with Inoperable Pancreatic Malignancy (PANcreatic Cancer Dietary Assessment—PanDA)

Lindsay Carnie, Dinakshi Shah, Kate Vaughan, Zainul Abedin Kapacee, Lynne McCallum, Marc Abraham, Alison Backen, Mairéad G McNamara, Richard A Hubner, Jorge Barriuso, Loraine Gillespie, Angela Lamarca and Juan W. Valle

1. Supplementary Material S1

1.1. Demographic cohort

1.1.1. Eligibility

Eligible patients with biopsy-proven or clinically-suspected (specialist multidisciplinary team meeting) aPC (locally-advanced or metastatic) being considered for first-line chemotherapy. Patients diagnosed with PanNET may have had received previous systemic treatment, but not be on active treatment at time of study entry. Patients with previous gastric, duodenal, or pancreatic resection or intolerance to pork-containing products or who declined taking such products for religious or personal reasons were excluded. Patients were also not eligible if comorbidities that increase the risk of PEI are present, described in the protocol write up. [29]

1.1.2. Clinical assessments

A single dietetic assessment (weight, body mass index (BMI), Mid-Upper Arm Circumference (MUAC), handgrip strength and stair climb test (SC-test)), full nutritional blood panel from standard of care bloods (haemoglobin, mean corpuscular volume, international normalised ratio, serum total protein concentration, albumin, pre-albumin, retinol binding protein, cholesterol (including total, high-density lipoprotein [HDL] and low-density lipoprotein [LDL]), triglycerides, amylase, vitamins A, D and E, glycated haemoglobin (HbA1c), transferrin, ferritin, magnesium, lymphocytes, neutrophils) and PEI symptom assessment was performed [29]. FE-1 was performed if clinically indicated. The Functional Assessment of Anorexia Cachexia Tool (Anorexia Cachexia Scale) with Visual Analogue Scale (FAACT–A/CS (with VAS)) assessed for anorexia. Assessment of ongoing or need for PERT also took place. Additional visits could be scheduled as clinically indicated. Data on anti-cancer treatment received (including chemotherapy-dose intensity (**Supplementary Material S2**) and patient outcome was collected.

1.1.3. Primary objective

To prospectively assess the prevalence of PEI (defined as the proportion of patients with dietitian-assessed symptoms/findings in keeping with a PEI diagnosis) in patients with aPC.

1.1.4. Secondary objectives

Secondary objectives included ascertaining the prevalence of PEI-related symptoms, percentage of patients receiving PERT at baseline and assessing the nutritional status of patients (using nutritional blood panel, weight, BMI, MUAC, handgrip, SC-test FAACT–A/CS (with VAS).

1.2. Diagnostic cohort

1.2.1. Eligibility

In addition to the Demographic Cohort eligibility criteria, patients were required to be willing to undergo the ^{13}C-MTBT and FE-1 tests (additional visit). Patients allergic to metoclopramide, a prokinetic used in the ^{13}C-MTBT, were excluded. To facilitate recruitment to this cohort, patients with potentially-operable disease who were not planning to undergo surgery (i.e. co-morbidities) and patients diagnosed with adenocarcinoma (and variants) who had received previous systemic treatment but had no active treatment for at least 3 months were eligible (Version 8, approved 21st April 2020).

1.2.2. Clinical assessments

Further to the clinical assessments performed for the Demographic Cohort, ^{13}C-MTBT and FE-1 were completed. An acceptability questionnaire was completed after the ^{13}C-MTBT and FE-1. The ^{13}C-MTBT was provided by Seahorse Laboratories Ltd. and analysed by Isoanalytical Limited; fasting for 10 hours prior to the test was required, and 13 breath samples were collected every 30 minutes for six hours, (full protocol in **Supplementary Material 3**). As the ^{13}C-MTBT would be challenging for patients to tolerate, acceptability was assessed after 10 completed tests, prior to further recruitment.

1.2.3. Primary objective

To design the most informative screening panel to identify patients with aPC who were at risk of PEI. The ^{13}C-MTBT was utilised to characterise patients with or without PEI as the gold standard.

1.2.4. Secondary objectives

Additional to the demographic cohort secondary objectives, the feasibility and acceptability (using a specifically-designed questionnaire) of performing the ^{13}C- MTBT and FE-1 tests were assessed and the impact of screening panel results on OS (Demographic and Diagnostic cohorts jointly).

1.3. Follow-up cohort

1.3.1. Eligibility

If follow-up oncology visits were planned alongside the criteria for the diagnostic cohort (except the ^{13}C-MTBT), and if patients were willing to complete the screening panel assessments and follow-up visits.

1.3.2. Clinical assessments

Following interim analysis on 02-June-2020, the study protocol was amended reflecting the required assessments for the Follow-up Cohort; version 9, 18th August 2020. Thus, patients in this cohort needed to complete a dietitian-led baseline assessment (weight, BMI, MUAC, handgrip strength and FAACT–A/CS (with VAS)), concomitant treatment (PEI-directed), PS and comorbidities), nutritional panel (standard of care bloods, including vitamin A and D, and magnesium if possible) and QoL questionnaires (QLQ-C30 and QLQ-PAN26). The screening panel result (MUAC and FE-1) classified patients as high or low-medium risk of PEI and dietetic assessment aimed to confirm the presence/absence of PEI. PERT was initiated, as required, and dose titrated following a pre-specified protocol (**Supplementary Material 4**). In addition, follow-up dietetic assessment occurred at week 6 (+/- 2 weeks), month 3 (+/- 3 weeks) and month 6 (+/- 3 weeks). Feedback questionnaires assessing views of dietetic input and screening panel acceptability were completed between weeks 4-6. Further reviews completed to reassess symptoms/PERT, if clinically indicated.

1.3.3. Primary objective

To evaluate the performance of the designed screening panel, in a prospective cohort of patients with aPC.

1.4. Secondary objectives

To evaluate the feasibility of using the designed screening panel (percentage of patients able to complete it), the agreement between the screening panel and dietetic assessment on the presence of PEI, the acceptability of the screening panel and the perception of dietetic care provided by patients. PERT compliance and adverse events associated with PERT were assessed (by recording the worst toxicity grade over all cycles according to the common terminology criteria for adverse events (CTCAE) version 4.3[30]), together with longitudinal changes to nutritional status (BMI, weight, MUAC and handgrip strength), symptoms, screening panel results, anorexia score and QoL scores. Finally, the impact of dietetic interventions on OS, chemotherapy starting rate and dose intensity (**Supplementary Material S2**) were explored.

2. Supplementary Material S2: chemotherapy intensity calculation guidelines

2.1. Calculating chemotherapy dose

- Calculate 'dose due' up to cycle 3 (6 for FOLFIRINOX)/interval scan if:
    - Patient RIP after cycle 1 of chemotherapy
    - Chemotherapy stopped due to poor PS
    - Chemotherapy stopped due to toxicity
- Calculate 'dose due' until cycle 6 (12 for FOLFIRINOX)/end of treatment scan if:
    - Chemotherapy stopped after cycle 4 for any reason above
- Calculate 'dose due' until last cycle of chemotherapy received if:
    - If patient choice to stop chemotherapy
    - Treatment stopped due to progression
    - If chemotherapy put on hold due to COVID 19
- If chemotherapy continues beyond 6 cycles (e.g. PRIMUS), calculate until death
- If patient is still undergoing chemotherapy (e.g. cycle 34), calculate up until date of data analysis
- If a day of chemotherapy omitted, use '0' for the 'dose received' that day
- If a dose delay (e.g. by a week), this was included, however if omitted completely = '0% received'
- If patient started one chemotherapy and then switched after one cycle to another, work out 'dose received' separately and add together
- If part of chemotherapy combination (e.g. capecitabine) stopped due to toxicity, calculate 'dose due' as if it should be received up until next scan due
- SSA – do not work out dose intensity

2.2. Dose reductions

- If reduced due to toxicity/bloods, keep 'dose due' the same
- If reduced due to a change in weight, change 'dose due' to new amount

2.3. Scans

- If no scan completed, did patient progress = 'no'. Put date of death as 'date of progression'
- If progressed after 1st line chemotherapy, included in 'did the patient progress' & date of scan
- If response was seen with 2nd line treatment do not include as the 'best radiological response'

Abbreviations: SSA, somatostatin analogue; PS, performance status; RIP, rest in peace.

3. Supplementary material S3: breath test protocol (©Seahorse Laboratories Ltd. 2016)

How the breath test works

1,3-distearyl-2-{carboxyl-^{13}C}octanoylglycerol, the so called ^{13}C-Mixed Triglyceride (Seahorse Laboratories Limited, UK) is a breath test devised to measure PEI. It is a non-invasive method to obtain information about the digestion of consumed lipids; this in turn is informative about the exocrine enzyme activity of the pancreas (specifically pancreatic lipase). The test components are supplied by Seahorse Laboratories Limited, UK.

3.1. Applications of 13C-Mixed Triglyceride Breath Test

The ^{13}C-Mixed Triglyceride Breath Test assesses duodenal pancreatic lipase activity. It is therefore useful for the investigation of severe exocrine pancreatic insufficiency. If applied under strict conditions even mild to moderate forms can be assessed with high sensitivity and specificity. The patient should have fasted for 10 hours prior to the test. The patient must not drink carbonated water or soft drinks prior to the test since that might interfere with the results. In addition oxygen supplementation should be avoided because increased oxygen content in exhaled breath can influence $^{13}CO_2$ measurement by NDIRS.

3.2. Metabolic Principle

1,3-distearyl-2-{carboxyl-^{13}C}octanoylglycerol, the so called ^{13}C-Mixed Triglyceride passes through the stomach and is digested by lipase activity in the duodenum. The two distearyl groups have to be hydrolysed by pancreatic lipase before absorption and metabolism of the ^{13}C-octanoyl monoglyceride. Thus, the oxidation to $^{13}CO_2$ is dependent on the rate-limiting step of hydrolysis of the fatty acids in positions 1 and 3.

3.3. Preparing patients for testing

It is important that patients follow these guidelines prior to taking the test. These will be clearly explained in the Patient Information Sheet;

3.3.1. Fasting

Patients must fast (not eat or drink) for at least 10 hours (overnight), before taking the test.

3.3.2. Diet

Patients must avoid eating foods rich in naturally-occurring ^{13}C for **48 hours before** taking the breath test. During the 48 hours before the test, patients must exclude broccoli or any food containing corn (corn bread, popcorn, cornflakes etc.) from their diet.

3.3.3. Drinking during the test

Patients may only consume a total of 250ml of still water during the six hour test (not fizzy water which would contain CO_2). This can either be all drunk at once at the start of the test, or consumed in smaller portions during the six hour test; drinking more liquid than 250ml will invalidate the test result.

3.3.4. Tobacco consumption

Patients must not smoke during the overnight fasting time, or during the test.

3.3.5. Medication

Treatment for any chronic condition should continue as normal before and during the test. However, PERT (Pancreatic Enzyme Replacement Therapy) such as CREON should be avoided if possible (to avoid confounding breath test results) and taken after the test is completed.

3.3.6. Diabetic patients

The doctor in charge should decide on the on the best way for a patient with diabetes to manage their condition during this test, since they might be at risk of hypoglycemia due to the overnight fasting required. Patients are advised to avoid taking diabetic medication until after the test to avoid risk of hypoglycaemia during the fasting period.

3.3.7. Antibiotics

It is advisable to take the test once antibiotics treatment has finished.

3.3.8. Proton pump inhibitors

Patients occasionally taking proton pump inhibitors (omeprazole or similar) are advised to stop the treatment on the day the test is taken until the test is completed.

3.3.9. Test materials and methods

The following items are provided Seahorse Laboratories Limited for carrying out this test:

- 150mg of ^{13}C-mixed triglycerides
- 13 bags to collect the breath samples (1 baseline sample, one at each of 30, 60, 90, 120, 150, 180, 210, 240, 270, 300, 330 and 360 minutes).
- A mouthpiece to introduce the breath into the collection bags.

3.3.10. Conducting the breath test

The test will be carried out in the hospital, **by the research dietitian** and will last around 6 hours.

Participants will have their height and weight recorded prior to the breath test. If these measurements are available from previous visits, repeating them at time of breath test will not be mandatory with the exception of: 1) patients who had such measurement taken more than 2 weeks ago or 2) significant weight loss from previous measurement is reported by the patient.

Additional items required (not included by Seahorse Laboratories Limited);

- 1 Metoclopramide tablet (10mg)
- Butter (weight of butter calculated at 0.25g of butter per Kg body weight)
- Two slices of bread (standard white sliced bread) approximately 100g in weight
- 250ml of still water

1. The patient will take the Metoclopramide tablet (to encourage stomach emptying), 20-30 minutes before the test food intake.
2. The dietitian will spread the weighed butter onto the two slices of bread. Weight of butter will be calculated at 0.25g of butter per Kg body weight.
3. The dietitian will sprinkle the 150mg of ^{13}C-mixed triglycerides over the butter spread on the slices of bread.
4. The dietitian will then collect the baseline breath sample from the patient (breath into the bag using the mouthpiece provided). The patient should hold his/her breath as long as possible so as to ensure maximum CO_2 concentration in the breath, then blow as hard as possible into the bag until the lungs are empty.
5. The dietitian will remove the mouthpiece and immediately insert the cap on the bag firmly (ensuring a firm seal).
6. The patient will then consume the test food (prepared bread and butter), along with some of the water. The patient may consume all the water at this point (but no other liquid is allowed during the 6 hour test, so the patient may wish to save some for later). The dietitian should note the time.
7. Breath samples will be collected in the same manner at 30, 60, 90, 120, 150, 180, 210, 240, 270, 300, 330 and 360 minutes after the baseline sample. The dietitian should

ensure that all bags are correctly labeled with the patient's trial number, date of birth, height, weight, gender and sample time point.

8. After six hours, the patient has completed their participation in this test. If the patient does not wish to proceed beyond 4 hours, the test can be stopped at this point (however continuing up to completion of 6 hours is encouraged).
9. The dietitian should then arrange the collection of the samples by Iso-Analytical where the breath samples will be analysed for exhaled CO_2;

Iso-Analytical Limited,
The Quantum, Phase 3
Marshfield Bank Business Park,
Crewe, Cheshire, CW2 8UY,
United Kingdom (Iso-Analytical Limited)
Telephone: 01270 509533
Fax: 01270
Email: info@iso-analytical.com

10. Iso-Analytical will return the results to the hospital within 5 days of receiving the samples. Results of breath tests are expected to be received 2-4 weeks after the test taking place.
11. Based on results provided, the final results of breath test will be calculated following pre-defined SOPs.

Patients will be contacted with results of breath test and treatment amended only if clinically required.

There will be no changes to patient management based only on breath test results.

3.3.11. Results

Patients who have **cumulative ^{13}C dose** values **below 29%** (after the six hour test) are considered to have a lipid digestion disorder due to exocrine pancreatic insufficiency, based on a shortage of lipase.

Patients who have **cumulative ^{13}C dose** values **above 29%** (after the six hour test) are considered to be healthy in terms of lipid digestion and do not have exocrine pancreatic insufficiency based on a shortage of lipase.

4. Supplementary material S4: PERT algorithm

Abbreviations: PEI, pancreatic exocrine insufficiency; PERT, pancreatic enzyme replacement therapy; PPI, proton pump inhibitor.

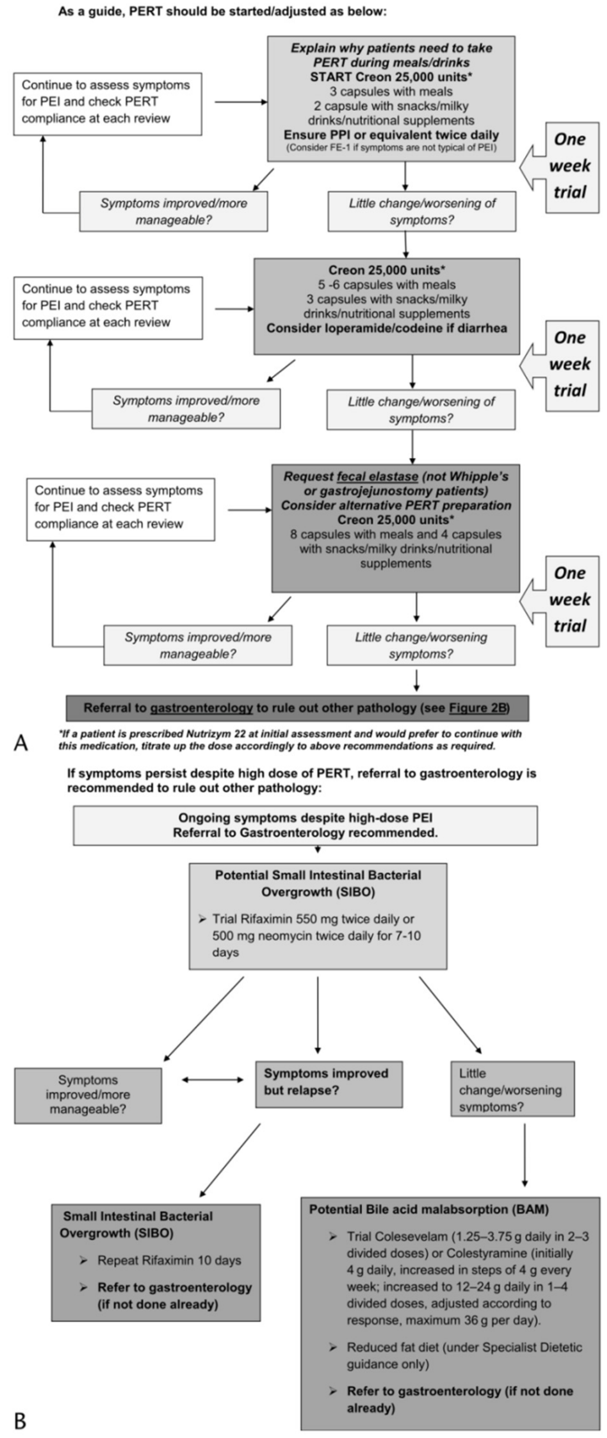

5. Supplementary material S5: Selection of variables for the design of the screening panel

The first step screened variables of interest used exact univariate logistic regression (rather than logistic regression due to small sample size). Variables of interest were those meeting any of the following: OR ≥3 (risk factor for breath test-defined PEI), OR ≤0.3 (protective factor for breath test-defined PEI) or significant p-value (regardless of OR). Dichotomised variables were prioritised over continuous counterparts, if similarly informative.

The variables selected with the exact logistic regression were then included in the second step. For clinical variables, this was performed utilising a pre-model "allsets" Stata command. The pre-model with highest AUC was selected; if more than one pre-model

existed with similarly high AUC, the model with best Sensitivity and Specificity and the most significant p-value was chosen. For laboratory-based results, the preselected measurements of potential interest identified with exact univariate logistic regression, that were likely to provide similar information (i.e. liposoluble vitamins: Vitamin A and Vitamin D) were tested against each other, utilising multivariable exact logistic regression to choose the most informative (the one with highest OR in exact logistic regression or the dichotomised variable, if similar OR).

The pre-defined clinical and laboratory-based variables of interest were finally included in a joined "allset" model to identify the variables of most interest that would be included in the screening score. The same criteria described for selection of clinical variables of interest when using "allsets" were utilised in this final step.

6. Supplementary material S6: Consort diagrams

Demographic cohort

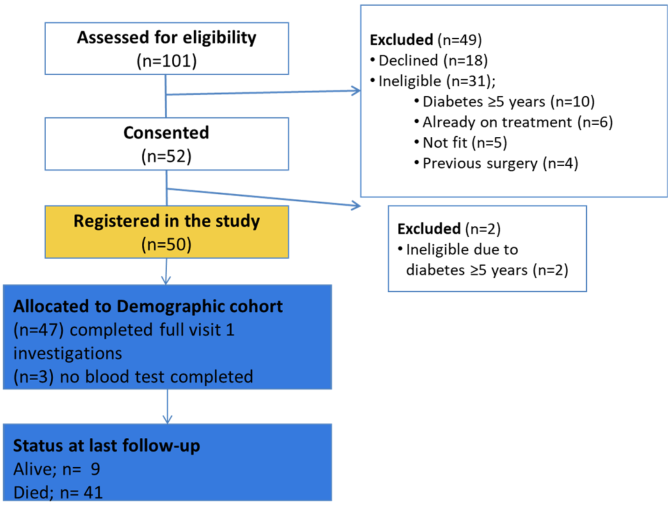

Diagnostic cohort

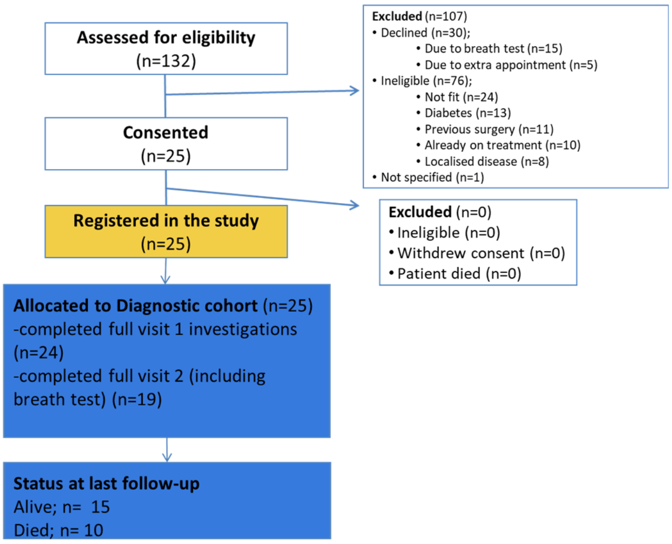

Follow-up cohort

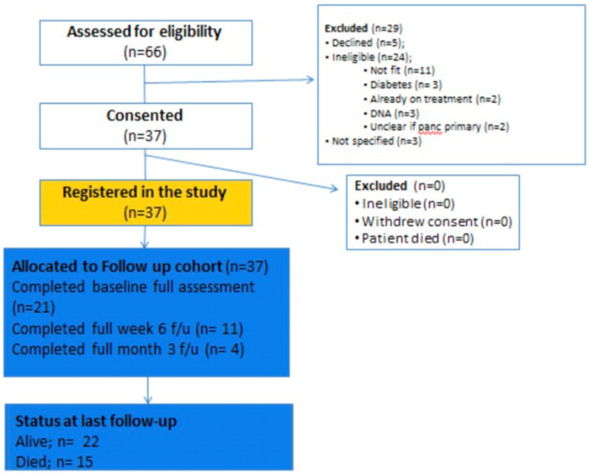

7. Supplementary material S7: Full details on dietitian and nutritional blood test assessment

Abbreviations: BMI, body mass index; ECOG-PS, Eastern Cooperative Oncology Group Performance Status; FAACT A/CS, functional assessment of anorexia/cachexia therapy anorexia/cachexia subscale; MUAC, mid-upper arm circumference; NET, neuroendocrine tumour; PEI, pancreatic exocrine insufficiency; PERT, pancreatic enzyme replacement therapy; PPI, proton pump inhibitor; VAS, visual analogue scale.

		Demographic cohort (n=50)		Diagnostic cohort (n=25)		P-value Dem vs Diagnostic cohort (Excluding missing)	Joined both cohorts (n=75)		Follow up cohort low risk screening panel		Follow up cohort high risk screening panel		P-value low vs high screening panel (excluding missing)	Full follow up cohort (n=37)	
PEI/PERT assessment at study entry		n	%	n	%	Ttest/Chi Squ p-value	n	%	N	%	n	%	Ttest/ChiSqu p-value	n	%
PEI present at study entry	No	17	34.0	9	36.0	0.864	26	34.67	1	14.29	3	10.34	1.000	4	12.81
	Yes	33	66.0	16	64.0		49	65.33	5	71.43	20	68.97		26	70.27
	Not assessed								1	14.29	6	20.69		7	18.92
PEI grade	Grade 1	12/33	36.36	8/16	50.0	0.674	20/49	26.67	5	71.43	8	27.59	n/a	13	35.14
	Grade 2	17/33	51.52	7/16	43.75		24/49	32.00	0	0.00	12	41.38		13	35.14
	Grade 3	4/33	12.12	1/16	6.25		5/49	6.67	0	0.00	0	0.00		0	0.00
	Not assessed								2	28.57	9	31.03		11	29.73
PEI diagnosis based on	Symptoms	33/33	100.0	13/16	81.25	0.030 (n/a)	46/49	93.88						1	2.70
	Symptoms + Faecal	0/33	0.0	3/16	18.75		3/49	6.12						25	67.57

	**elas-tase/breath test**														
	**No PEI/not assessed**												n/a	(4/7) 11	29.73
**PERT at time of or following first visit**	**No**	15	30.0	11	44.0	0.230	26	34.67	1	14.29	4	13.79	1.000	5	13.51
	**Yes**	35	70.0	14	56.0		49	65.33	5	71.43	19	65.53		25	67.57
	**Not assessed**								1	14.29	6	20.690		7	18.92
**PERT started by dietitian at study entry *patients with PEI not on PERT at study entry**	**No**	41	82.0	22	88.0	1.000	63	84.00	4	54.14	17	58.62	1.000	22	59.46
	**Yes***	6*	12.0	3*	12.0		9*	12.00	1	14.29	5	17.24		6	16.22
	**Not documented**	3	6.0	0	0.0		3	4.00	2	28.57	7	24.14		9	24.32
**PERT dose adjusted by dietitian at study entry *of these, 33 were pts with PEI, on 2 no PEI and PERT stopped **patients with PEI**	**No**	12	24.0	6	24.0	0.945	18	24.00	3	42.86	17	58.62	1.000	17	45.95
	**Yes**	23*	46.0	12	48.0		35*	46.67	2	28.57	5	17.24		10**	27.03
	**Not applicable**	15	30.0	7	28.0		22	29.33	2	28.57	7	24.14		10	27.03
**Known to dietitian at study entry *of these, 41 had PEI; 41/49 of pts (83.67%) with PEI were not known to dietitian at time of referral**	**No**	44	88.0	21	84.0	0.723	65* (41 with PEI, 24 with no PEI)	86.67	3	42.86	14	48.28	0.669	18	48.65
	**Yes**	6	12.0	4	16.0		10 (8 with PEI, 2 with no PEI)	13.33	3	42.86	9	31.03		12	32.43
	**Not assessed**								1	14.29	6	20.69		7	18.92
**Patient on PPI**	**No**	20	40.0	9	36.0	0.737	29	38.67	1	14.29	12	41.38	0.183	13	35.14
	**Yes**	30	60.0	16	64.0		46	61.33	5	71.43	11	37.93		17	45.95
	**Not assessed**								1	14.29	6	20.69		7	18.92
							**PEI symptoms at study entry**								
**PEI symptoms assessed (dietitian assessment)**	**Yes**	50	100.0	25	100.0	n/a	75	100.00	6	85.7	23	79.31	1.000	30	81.03
	**No (*too unwell)**								1	14.2	6	20.69		*7	18.92
**Symptoms in keeping with PEI**	**Yes**	33	66.0	16	64.0	0.864	49	65.33	5	71.4	20	68.97	1.000	26	70.27

Steatorrhea	Yes	24	48.0	10	40.0	0.512	34	45.33	2	28.57	13	44.83	0.674	16	43.24
	Grade 1	10	-	5	-	0.457	15	-	2	-	7	-		10	-
	Grade 2	12	-	3	-		15	-	0	-	5	-		5	-
	Grade 3	2	-	2	-		4	-	0	-	0	-		0	-
Weight loss	Yes	45	84.0	18	72.0	0.221	60	80.00	6	85.71	20	68.97	0.645	27	72.97
Absolute amount of weight loss (kg)	Median (range) (95% CI)	11.1 (0-38.1) (10.00-15.24)		7.85 (1.5-25.4) (6.19-11.81)		0.0920	10.1 (0-38.1) (9.47-13.47)		5.5 (3-14.8) (1.76-10.94)		12.7 (1-35.5) (9.29-17.19)		0.075	10.75 (1-35.5) (8.33-14.75)	
Relative amount of weight loss (%)	Median (range) (95% CI)	14.85 (0-32) (12.20-17.20)		10.25 (2.4-19.37) (8.17-13.57)		0.0646	13.45 (0-32) (11.58-15.43)		5.3 (4.2-16) (2.65-12.18)		18.5 (1.4-37.2) (11.96-20.98)		0.041	13 (1.4-37.2) (10.63-18.03)	
Time period weight loss identified (months)	Median (range) (95% CI)	4 (0-20) (4.01-6.75)		2 (1-24) (1.37-6.18)		0.2130	3 (0-24) (3.70-6.06)		3 (2-7) (1.49-6.17)		4 (1-12) (3.47-6.82)		0.418	3.5 (1-12) (3.38-6.04)	
Flatus indigestion	Yes	42	84.0	21	84.0	1.000	63	84.00	5	71.4	20	68.97	1.000	26	70.27
	Grade 1	24	-	12	-		36	-	5	-	12	-		18	-
	Grade 2	13	-	3	-		16	-	0	-	2	-		2	-
	Grade 3	5	-	6	-		11	-	0	-	1	-		1	-
Abdominal discomfort	Yes	25	50.0	11	44.0	0.624	36	48.00	5	71.4	13	44.83	0.402	19	51.35
	Grade 1	11	-	6	24.00		17	-	4	-	7	-		12	-
	Grade 2	12	-	4	16000		16	-	0	-	2	-		2	-
	Grade 3	2	-	1	4.00		3	-	0	-	1	-		1	-
Bowel movements prior to diagnosis	Frequency <1/day	10	20.0	7	28.0	0.762	17	22.67	0	0.00	5	17.24	0.672	6	16.22
	Frequency 1/day	29	58.0	12	48.0		41	54.67	4	57.14	14	48.28		18	48.65
	Frequency 2-3/day	10	12.0	6	24.0		16	21.33	2	28.57	2	6.90		4	10.81
	Frequency >3/day	1	10.0	0	0.0		1	1.33	0	0.00	2	6.90		2	5.41
Bowel movements prior to diagnosis	Type 1	0	0.0	1	4.0	0.038	1	1.33	1	14.29	0	0.00	0.372	1	2.70
	Type 2	1	2.0	3	12.0		4	5.33	0	0.00	5	17.24		6	16.22
	Type 3	10	20.0	6	24.0		16	21.33	2	28.57	6	29.69		8	21.62
	Type 4	32	64.0	9	36.0		41	54.67	2	28.57	8	27.59		10	27.03
	Type 5	4	8.0	1	4.0		5	6.67	0	0.00	1	3.45		1	2.70
	Type 6	3	6.0	4	16.0		7	9.33	1	14.29	1	3.45		2	5.41
	Type 7	0	0.0	1	4.0		1	1.33	0	0.00	2	6.90		2	5.41
	Not assessed								1	14.29	6	20.69		7	18.92
Bowel movements prior to diagnosis	Type 1-5	47	94.0	20	80.0	0.064	67	89.33	5	71.43	20	68.97	1.000	26	70.27
	Type 6-7	3	6.0	5	20.0		8	10.67	1	14.29	3	10.34		4	10.81

**Bowel movements at time of study entry**	**Frequency <1/day**	19	38.0	10	40.0	0.232	29	38.67	0	0.00	5	17.24	0.300	6	16.22
	**Frequency 1/day**	14	28.0	10	40.0		24	32.00	2	18.57	7	24.14		9	24.32
	**Frequency 2-3/day**	10	20.0	5	20.0		15	20.00	3	42.87	7	24.14		10	27.03
	**Frequency >3/day**	7	14.0	0	0.0		7	9.33	1	14.29	2	6.90		3	8.11
**Bowel movements at time of study entry**	**Type 1**	5	10.0	0	0.0	0.501	5	6.67	1	14.29	3	10.34	0.657	4	10.81
	**Type 2**	1	2.0	0	0.0		1	1.33	0	0.00	2	6.90		2	5.41
	**Type 3**	4	8.0	4	16.0		8	10.67	1	14.29	1	6.90		3	8.11
	**Type 4**	17	34.0	11	44.0		28	37.33	4	57.14	8	27.59		12	32.43
	**Type 5**	6	12.0	2	8.0		8	10.67	0	0.00	0	0.00		0	0.00
	**Type 6**	7	14.0	5	20.0		12	16.00	0	0.00	5	17.24		6	16.22
	**Type 7**	10	20.0	3	12.0		13	17.33	0	0.00	3	10.34		3	8.11
	**Not assessed**								1	14.29	6	20.60		7	18.92
**Bowel movements at time of study entry**	**Type 1-5**	33	66.0	17	68.0	0.862	50	66.67	6	85.71	15	51.72	0.148	21	56.76
	**Type 6-7**	17	34.0	8	32.0		25	33.33	0	0.00	8	27.59		9	24.32
**Other**	**Pain**	1	2.0	2	8.0	n/a	3	4.00	0	-	1	-	0.148	21	56.75
	**Early satiety**	1	2.0	0	0.0	n/a	1	1.33	0	-	0	-		9	24.32
**Dietitian assessment**															
**Height (cm)**	**Median (range) (95% CI)**	168.5 (145-190) (165.52-171.12)		168 (155-194) (165.59-173.89)		0.5610	168 (145-194) (166.52-171.07)		163 (146.8-178.7) (150.88-172.84)		167 (151.8-190.9) (165.06-172.64)		0.1136	167 (146.8-190.9) (163.91-170.87)	
**Weight (kg)**	**Median (range) (95% CI)**	69.43 (42.4-103.85) (66.15-74.58)		73.5 (51.4-105.7) (66.97-80.10)		0.3971	71 (42.4-105.7) (67.93-74.91)		77.25 (60.4-100.9) (62.79-89.20)		66 (44.1-92.4) (60.71-69.75)		0.0458	66.1 (44.1-100.9) (62.99-71.48)	
**BMI (kg/m2)**	**Median (range) (95% CI)**	24.38 (15.67-35.84) (23.44-26.36)		24.23 (20.04-39.52) (23.58-27.17)		0.6942	24.38 (15.67-39.52) (23.94-26.18)		28.03 (23.58-33.76) (25.66-32.15)		22.96 (16.70-31.59) (21.29-23.97)		0.0001	23.43 (16.70-33.75) (22.46-25.29)	
	**Low (<18.5)**	4	8.0	0	0.0	0.294	4	5.33	0	0.00	3	10.34	1.000	3	8.11

**FAACT questionnaire completed**	**Yes**	50	100.0	25	100.0	n/a	75	100.00	6	85.71	27	93.10	0.488	34	91.89
**Low appetite (based on FAACT-A/C-S)**	**Yes (based on points +/- vas)**	37	74.0	16	64.0	0.370	53	70.67	5	71.43	23	79.31	1.000	29	78.38
	**Yes; FAACT <=37 points**	36	72.0	16	64.0	0.479	44	69.33	5	71.42	20	68.97	1.000	26	70.27
	**No ; FAACT >37 points**	14	28.0	9	36.0		31	30.67	1	14.29	7	24.14		8	21.62
	**Yes; VAS <=7 cm**	31	62.0	13	52.0	0.407	52	58.67	4	57.14	16	55.17	1.000	21	56.76
	**No ; VAS>7 cm**	19	38.0	12	48.0		23	41.33	2	28.57	9	31.03		11	29.73
**MUAC (cm)**	**Low (<=P25) (2 points in screening panel)**	26	52.0	13	48.0	0.931	39	52.00	0	0.00	29	100.00	n/a	29	78.38
	**Normal (>P25) (0 points in screening panel)**	23	46.0	12	52.0		35	46.67	7	100.00	0	0.00		7	18.92
	**Not done**	1	2.0	0	0.0		1	1.33	0	0.00	0	0.00		1	2.70
**Handgrip (kg)**	**Low (<median 83.6)**	31	62.0	6	24.0	0.002	37	49.33	4	57.14	10	65.52	0.394	14	37.84
	**Strong (>=median 83.6)**	19	38.0	19	76.0		38	50.67	3	42.86	19	34.48		22	59.46
	**Not done**								0	0.00	0	0.00		1	2.70
**SC test (watts)**	**Slow (<median 217.95)**	21	42.0	16	64.0	0.030	37	49.33	-	-	-	-	-	-	-

	**Quick (>=median 217.95)**	23	46.0	5	20.0		28	37.33	-	-	-	-	-	-	-
	**Not well enough to complete**	6	12.0	4	16.0		10	13.33	-	-	-	-	-	-	-
**Breath test (n=19)**	**PEI (<=29)**	-	-	7/19	36.84	n/a	-	-	-	-	-	-	-	-	-
	**Abnormal (>29)**	-	-	12/19	63.16	n/a	-	-	-	-	-	-	-	-	-
	**Median result (range) (95 % CI)**			31 (6.79-56.41) (25.31 - 36.81)		n/a			-	-	-	-	-	-	-
**Faecal elastase**	**PEI (<200)**	1/2	50.	12/20	60.0	n/a	13/22	59.09	5	71.43	12	41.38	n/a	17	45.95
	**No PEI (>=200)**	1/2	50.0	8/20	40.0	n/a	9/22	40.91	1	14.29	6	20.69		7	18.92
	**Not performed**	48	-	5	-	n/a	53	-	1	14.29	11	41.38		12	32.43
	**Median result (range) (95 % CI)**	260.5 (21-500) (0-3303.67)		148.5 (15-500) (97.42 - 256.28)		n/a	148.5 (15-500) (104.97-263.93)		15 (15-428) (0-260.78) 6 observations		50.5 (15-500) (71.04-262.40) 18 observations		0.3579	21.5 (15-500) (67.13 - 224.87) 24 observations	
**Nutritional blood panel**															
**Blood panel performed**	**No**	3	6.0	1	4.0	1.000	4	5.33	0	0.00	1	3.45	1.000	1	2.70
	**Yes**	47	94.0	24	96.0		71	94.67	7	100.00	28	96.55		36	97.30
**Ferritin**	**Normal**	31	62.0	12	48.0	0.422	43	57.33	-	-	-	-	-	-	-
	**Low**	1	2.0	1	4.0		2	2.67	-	-	-	-	-	-	-
	**High**	15	30.0	10	40.0		25	33.33	-	-	-	-	-	-	-
	**Not done**	3	6.0	2	8.0		5	6.67	-	-	-	-	-	-	-
**LDL**	**Normal**	25	50.0	17	68.0	0.358	42	56.00	-	-	-	-	-	-	-
	**High**	17	34.0	7	28.0		24	32.00	-	-	-	-	-	-	-
	**Not done**	8	16.0	1	4.0		9	12.00	-	-	-	-	-	-	-
**Vitamin D**	**Normal**	24	48.0	11	44.0	0.615	35	46.67	3	42.86	17	58.62	0.410	21	56.76
	**Low**	22	44.0	13	52.0		35	46.67	4	57.14	10	34.48		14	37.84
	**Not done**	4	8.0	1	4.0		5	6.67	0	0.00	2	6.90		2	5.41
**Total cholesterol**	**Normal**	23	46.0	14	56.0	0.453	37	49.33	-	-	-	-	-	-	-
	**High**	24	48.0	10	40.0		34	45.33	-	-	-	-	-	-	-
	**Not done**	3	6.0	1	4.0		4	5.33	-	-	-	-	-	-	-

**Triglycerides**	**Normal**	41	82.0	22	88.0	1.000	63	84.00	-	-	-	-	-	-	-
	**High**	4	8.0	2	8.0		6	8.00	-	-	-	-	-	-	-
	**Not done**	5	10.0	1	4.0		6	8.00	-	-	-	-	-	-	-
**HBA1C**	**Cont. variable (median) (range) (95% CI)**	41.5 (30-109) (40.49-50.46)		41 (29-67) (38.33-46.84)		0.4344	41 (29-109) (40.96-47.96)		41 (28-64) (19.11-67.89)		40 (25-133) (32.74-60.86)		0.8093	40 (25-133) (34.49-56.01)	
**Vitamin E**	**Normal**	44	88.0	23	92.0		67	89.33	7	100.00	26	89.66	n/a	34	91.89
	**Low**	2	4.0	0	0.0	0.549	5	2.67	0	0.00	0	0.00		0	0.00
	**Not done**	4	8.0	2	8.0		6	8.00	0	0.00	3	10.34		3	8.11
**Vitamin A**	**Normal**	37	74.0	18	72.0	0.832	55	73.33	6	85.71	17	58.62	0.397	23	62.16
	**Low**	9	18.0	5	20.0		14	18.67	1	14.29	9	31.03		11	29.73
	**Not done**	4	8.0	2	8.0		6	8.00	0	0.00	3	10.34		3	8.11
**Amylase**	**Normal**	31	62.00	16	64.00	1.000	47	62.67	-	-	-	-	-	-	-
	**Low**	13	26.00	6	24.00		19	25.33	-	-	-	-	-	-	-
	**High**	3	6.00	2	8.00		5	6.67	-	-	-	-	-	-	-
	**Not done**	3	6.00	1	4.00		4	5.33	-	-	-	-	-	-	-
**Magnesium**	**Normal**	42	84.00	23	92.00	0.164	65	86.67	6	85.17	26	89.66	0.374	32	86.49
	**Low**	5	10.00	0	0.00		5	6.67	1	14.29	1	3.45		3	8.11
	**Not done**	3	6.00	2	8.00		5	6.67	0	0.00	2	6.90		2	5.41
**Transferrin**	**Normal**	22	44.00	14	56.00	0.280	36	48.00	-	-	-	-	-	-	-
	**Low**	20	40.00	7	28.00		27	36.00	-	-	-	-	-	-	-
	**Not done**	8	16.00	4	16.00		12	16.00	-	-	-	-	-	-	-
**INR**	**Normal**	13	26.00	5	20.00	0.500	18	24.00	3	42.86	6	20.69	0.295	9	24.32
	**High**	33	66.00	19	76.00		52	69.33	2	28.57	16	55.17		19	51.35
	**Not done**	4	8.00	1	4.00		2	6.67	2	28.57	7	24.14		9	24.32
**Hb**	**Normal**	35	70.00	21	84.00	0.152	56	74.67	6	85.71	21	72.41	1.000	27	72.97
	**Low**	14	28.00	3	12.00		17	22.67	1	14.29	7	24.14		9	24.32
	**Not done**	1	2.00	1	4.00		2	2.67	0	0.00	1	3.45		1	2.70
**MCV**	**Normal**	41	82.00	20	80.00	0.438	61	81.33	5	71.43	23	79.31	0.608	28	75.68
	**Low**	0	0.00	1	4.00		1	1.33	0	0.00	0	0.00		0	0.00
	**High**	8	16.00	3	12.00		11	14.67	2	28.57	5	17.24		8	21.62
	**Not done**	1	2.00	1	4.00		2	2.67	0	0.00	1	3.45		1	2.70
**Serum total protein**	**Normal**	48	96.00	22	88.00	0.250	70	93.33	7	100.00	25	86.21	1.00	33	89.19
	**Low**	1	2.00	2	8.00		3	4.00	0	0.00	3	10.34		3	8.11
	**Not done**	1	2.00	1	4.00		2	2.67	0	0.00	1	3.45		1	2.70
**Albumin**	**Normal**	45	90.00	23	92.00	1.000	68	90.67	6	85.71	27	93.10	0.365	33	89.19
	**Low**	4	8.00	1	4.00		5	6.67	1	14.29	1	3.45		3	8.11
	**Not done**	1	2.00	1	4.00		2	2.67	0	0.00	1	3.45		1	2.70
**Lymphocytes**	**Normal (>=1500)**	23	46.00	6	24.00	0.065	29	38.67	3	42.86	15	51.72	0.691	18	48.65

	**Low**	27	54.00	19	76.00		46	61.33	4	57.14	13	44.83		18	48.65
	**Not done**	0	0.00	0	0.00		0	0.00	0	0.00	1	3.45		0	0.00
**Neutrophils**	**Normal (2000-7500)**	36	72.00	21	84.00	0.316	57	76.00	6	85.71	20	68.97	0.648	27	72.97
	**Low**	1	2.00	1	4.00		2	2.67	0	0.00	0	0.00		0	0.00
	**High**	13	26.00	3	12.00		16	21.33	1	14.29	8	27.59		9	24.32
	**Not done**	0	0.00	0	0.00		0	0.00	0	0.00	1	3.45		1	2.70
**NLR**	**Low (below median; <4.25)**	26	52.00	11	44.00	0.514	37	49.33	4	57.14	13	44.83	0.684	17	45.95
	**High (above median; >=4.25)**	24	48.00	14	56.00		38	50.67	3	42.86	16	55.17		20	54.05
	**Not done**	0	0.00	0	0.00		0	0.00	0	0.00	0	0.00		0	0.00

8. Supplementary material S8: Evolution of bowel movement at time of study entry: 8A shows bowel movement type; 8B shows frequency.

Definition of stool type using the bristol stool chart; type 1 – separate hard lumps, type 2 – sausage-shaped and lumpy, type 3 – like a sausage with cracks on the surface, type 4 – smooth or soft like a sausage, type 5 – soft blobs with clear edges, type 6 – fluffy with ragged edges, type 7 – liquid [31].

A. Changes in type of bowel movements prior to diagnosis vs at study entry

N=75 (joint demographic and diagnostic cohorts)		At study entry						
		Type 1	Type 2	Type 3	Type 4	Type 5	Type 6	Type 7
Prior to diagnosis	Type 1	0	0	0	1	0	0	0
	Type 2	0	0	1	0	1	2	0
	Type 3	3	1	4	3	0	3	2
	Type 4	2	0	1	18	4	5	11
	Type 5	0	0	1	2	1	1	0
	Type 6	0	0	1	3	2	1	0
	Type 7	0	0	0	1	0	0	0

Orange: trend to more constipation→ 17/75= 22.67%
Green: stable frequency of bowel movements → 24/75= 32.00%
Red: worsening bowel movements towards more diarrhoea→ 34/75=45.33%

B. Changes in bowel movement frequency prior to diagnosis vs at study entry

N=75 (joined demographic and diagnostic cohorts)		At study entry			
		<1/days	1/day	2-3/day	>3/day
Prior to diagnosis	<1/day	11	5	0	1
	1/day	15	15	8	3
	2-3/day	3	3	7	3
	>3/day	0	1	0	0

Orange: trend towards more constipation→ 22/75= 29.33%
Green: stable type of bowel movements → 33/75= 44.00%
Red: worsening bowel movements towards more diarrhoea→ 20/75=26.67%

9. Supplementary material S9: Comparison of high risk and low risk populations in the follow up cohorts

Abbreviations: BMI, body mass index; ECOG-PS, Eastern Cooperative Oncology Group Performance Status; FAACT A/CS, functional assessment of anorexia/cachexia therapy anorexia/cachexia subscale; MUAC, mid-upper arm circumference; NET, neuroendocrine tumour; PEI, pancreatic exocrine insufficiency; PERT, pancreatic enzyme replacement therapy; PPI, proton pump inhibitor; VAS, visual analogue scale.

Grade defined as the severity of the adverse event as per CTCAE v5.0. Grade 1 – mild symptoms or asymptomatic with no interventional indicated. Grade 2 – moderate symptoms, limiting age-appropriate self-care and minimal intervention required. Grade 3 – severe symptoms but not immediately life-threatening, limiting self-care and hospitalisation required.

Patient characteristics		Low risk screening panel (n=7)		High risk screening panel (n=29)		P-value low vs high panel (excluding missing)	Full Follow-up cohort (n=37)	
		n	%	n	%	Ttest/ChiSqu/Fisher p-value	n	%
**Age at study entry**	**Median (range) (95% CI)**	69.91 (56.43-79.82) (63.77)		70.58 (44-85.11) (63-72)		0.6136	70.57 (44-85.11) (65-72)	
**Gender**	**Female**	5	71.43	11	37.92	0.204	17	45.95
	**Male**	2	28.57	18	62.07		20	54.05
**Comorbidities**	**None**	1	14.29	12	41.38	0.277	13	35.14
	**Mild**	4	57.14	14	48.28		18	48.65
	**Moderate**	2	28.57	2	6.90		5	13.51
	**Severe**	0	0.00	1	3.45		1	2.70
**Localisation primary pancreatic tumour**	**Head/neck**	5	71.43	14	48.28	0.844	20	54.05
	**Body**	1	14.29	9	31.03		10	27.03
	**Tail**	1	14.29	5	17.24		6	16.22
	**Not specified**	0	0.00	1	3.45		1	2.70
**Biopsy confirmed cancer**	**No**	0	0.00	0	0.00	n/a	0	0.00
	**Yes**	7	10.00	29	100.00		37	100.00
**Type of pancreatic cancer**	**Adenocarcinoma**	6	85.71	26	89.66	0.597	33	89.19
	**NET**	0	0.00	2	6.90		2	5.41
	**Other**	1	14.29	1	3.45		2	5.41
**Differentiation (if NET)**	**Grade 1**	0	-	1	-	n/a	1	-
	**Grade 2**	0	-	0	-		0	-
	**Grade 3**	0	-	1	-		1	-
	**Not NET**	7	-	27	-		35	-
**Ki 67 (if NET)**	**Median (range) (ki67)**	n/a	-	14 (1-27) (0-100)		n/a	14 (1-27) (0-100)	
**Functional (if NET)**	**Yes**	0	-	0	-	n/a	0	-
	**No**	0	-	2	-		2	-
	**Not NET**	7	-	27	-		35	-
**ECOG PS at study entry**	**0**	1	14.29	5	17.24	0.835	6	16.22
	**1**	4	57.14	17	58.62		21	56.76
	**2**	2	28.57	4	13.79		7	18.92
	**3**	0	0.00	3	10.34		3	8.11
	**4**	0	0.00	0	0.00		0	0.00
**Stage at study entry**	**Localised**	1	14.29	0	0.00	0.199	1	2.70
	**Locally advanced**	2	28.57	15	51.72		18	48.65
	**Metastatic**	4	57.14	14	48.28		18	48.65
**PEI/PERT assessment at study entry**								
**PEI present at study entry**	**No**	1	14.29	3	10.34	1.000	4	10.81
	**Yes**	5	71.43	20	68.97		26	70.27
	**Not assessed**	1	14.29	6	20.69		7	18.92
**PEI grade**	**Grade 1**	5	71.43	8	27.59	n/a	13	35.14
	**Grade 2**	0	0.00	12	41.38		13	35.14
	**Grade 3**	0	0.00	0	0.00		0	0.00
	**Not assessed**	2	28.57	9	31.03		11	29.73
**PEI diagnosis based on**	**Symptoms**					n/a	1	2.70
	**Symptoms + risk panel**						25	67.57
	**No PEI/not assessed**						(4/7) 11	29.73
	**No**	1	14.29	4	13.79	1.000	5	13.51

**PERT at time of or after first visit**	**Yes**	5	71.43	19	65.52		25	67.57
	**Not assessed**	1	14.29	6	20.69		7	18.92
**PERT started by dietitian at study entry *patients with PEI who were not on PERT at study entry**	**No**	4	57.14	17	58.62	1.000	22	59.46
	**Yes***	1	14.29	5	17.24		6	16.22
	**Not documented**	2	28.57	7	24.14		9	24.32
**PERT dose adjusted by dietitian at study entry *patients with PEI**	**No**	3	42.86	17	58.62	1.000	17	45.95
	**Yes**	2	28.57	5	17.24		10*	27.03
	**Not applicable**	2	28.57	7	24.14		10	27.03
**Already known to dietitian at study entry *of these, 41 had PEI; 41/49 of pts (83.67%) with PEI were not known to dietitian at time of referral**	**No**	3	42.86	14	48.28	0.669	18	48.65
	**Yes**	3	42.86	9	31.03		12	32.43
	**Not assessed**	1	14.29	6	20.69		7	18.92
**Patient on PPI**	**No**	1	14.29	12	41.38	0.183	13	35.14
	**Yes**	5	71.43	11	37.93		17	45.95
	**Not assessed**	1	14.29	6	20.69		7	18.92
**PEI symptoms at study entry (Dietitian assessment)**								
**PEI symptoms assessed (dietitian assessment)**	**Yes**	6	85.71	23	79.31	1.000	30	81.08
	**No**	1	14.29	6	20.69		7	18.92
**Symptoms in keeping with PEI**	**Yes**	5	71.43	20	68.97	1. 000	26	70.27
**Steatorrhea**	**Yes**	2	28.57	13	44.83	0.674	16	43.24
	**Grade 1**	2	-	7	-		10	-
	**Grade 2**	0	-	5	-		5	-
	**Grade 3**	0	-	0	-		0	-
**Weight loss**	**Yes**	6	85.71	20	68.97	0.645	27	72.97
**Absolute amount of weight loss (kg)**	**Median (range) (95% CI)**	5.5 (3-14.8) (1.76-10.94)		12.7 (1-35.5) (9.29-17.19)		0.0751	10.75 (1-35.5) (8.33-14.75)	
**Relative amount of weight loss (%)**	**Median (range) (95% CI)**	5.3 (4.2-16) (2.65-12.18)		18.5 (1.4-37.2) (11.96-20.98)		0.0412	13 (1.4-37.2) (10.63-18.03)	
**Time period over weight loss identified (months)**	**Median (range) (95% CI)**	3 (2-7) (1.49-6.17)		4 (1-12) (3.47-6.82)		0.4183	3.5 (1-12) (3.38-6.04)	
**Flatus indigestion**	**Yes**	5	71.43	20	68.97	1.000	26	70.27
	**Grade 1**	5	-	12	-		18	-
	**Grade 2**	0	-	2	-		2	-
	**Grade 3**	0	-	1	-		1	-
**Abdominal discomfort**	**Yes**	5	71.43	13	44.83	0.402	19	51.35
	**Grade 1**	4	-	7	-		12	-
	**Grade 2**	0	-	2	-		2	-
	**Grade 3**	0	-	1	-		1	-
**Bowel movements prior to diagnosis**	**Frequency <1/day**	0	0.00	5	17.24	0.672	6	16.22
	**Frequency 1/day**	4	57.14	14	48.28		18	48.65
	**Frequency 2-3/day**	2	28.57	2	6.90		4	10.81
	**Frequency >3/day**	0	0.00	2	6.90		2	5.41
**Bowel movements prior to diagnosis**	**Type 1**	1	14.29	0	0.00	0.372	1	2.70
	**Type 2**	0	0.00	5	17.24		6	16.22
	**Type 3**	2	28.57	6	20.69		8	21.62
	**Type 4**	2	28.57	8	27.59		10	27.03
	**Type 5**	0	0.00	1	3.45		1	2.70

	**Type 6**	1	14.29	1	3.45		2	5.41
	**Type 7**	0	0.00	2	6.90		2	5.41
	**Not assessed**	1	14.29	6	20.69		7	18.92
**Bowel movements prior to diagnosis**	**Type 1-5**	5	71.43	20	68.97	1.000	26	70.27
	**Type 6-7**	1	14.29	3	10.34		4	10.81
**Bowel movements at time of study entry**	**Frequency <1/day**	0	0.00	5	17.24	0.300	6	16.22
	**Frequency 1/day**	2	18.57	7	24.14		9	24.32
	**Frequency 2-3/day**	3	42.86	7	24.14		10	27.03
	**Frequency >3/day**	1	14.29	2	6.90		3	8.11
**Bowel movements at time of study entry**	**Type 1**	1	14.29	3	10.34	0.657	4	10.81
	**Type 2**	0	0.00	2	6.90		2	5.41
	**Type 3**	1	14.29	1	6.90		3	8.11
	**Type 4**	4	57.14	8	27.59		12	32.43
	**Type 5**	0	0.00	0	0.00		0	0.00
	**Type 6**	0	0.00	5	17.24		6	16.22
	**Type 7**	0	0.00	3	10.34		3	8.11
	**Not assessed**	1	14.29	6	20.69		7	18.92
**Bowel movements at time of study entry**	**Type 1-5**	6	85.71	15	51.72	0.148	21	56.76
	**Type 6-7**	0	0.00	8	27.59		9	24.32
**Worsening frequency bowel movements at time of study entry**	**Yes**	3	42.86%	7	24.14	0.370	10	27.03
**Worsening type bowel movements at time of study entry**	**Yes**	1	14.29%	8	27.59%	0.633	9	24.32
**Other**	**Pain**	0	-	1	-		1	-
	**Early satiety**	0	-	0	-		0	-
**Dietitian assessment**								
**Height (cm)**	**Median (range) (95% CI)**	163 (146.8-178.7) (150.88-172.84)		167 (151.8-190.9) (165.06-172.64)		0.1136	167 (146.8-190.9) (163.91-170.87)	
**Weight (kg)**	**Median (range) (95% CI)**	77.25 (60.4-100.9) (62.79-89.20)		66 (44.1-92.4) (60.71-69.75)		0.0458	66.1 (44.1-100.9) (62.99-71.48)	
**BMI**	**Median (range) (95% CI)**	28.03 (23.58-33.76) (25.66-32.15)		22.96 (16.70-31.59) (21.29-23.97)		0.0001	23.43 (16.70-33.75) (22.46-25.29)	
	**Low (<18.5)**	0	0.00	3	10.34	1.000	3	8.11
**FAACT questionnaire completed**	**Yes**	6	85.71	27	93.10	0.488	34	91.89
**Low appetite (based on FAACT-A/C-S)**	**Yes (based on points +/- vas)**	5	71.43	23	79.31	1.000	29	78.38
	**Yes; FAACT <=37 points**	5	71.43	20	68.97	1.000	26	70.27
	**No ; FAACT >37 points**	1	14.29	7	24.14		8	21.62
	**Yes; VAS <=7 cm**	4	57.14	16	55.17	1.000	21	56.76
	**No ; VAS>7 cm**	2	28.57	9	31.03		11	29.73
**MUAC**	**Low (<=P25) – 2 points in panel**	0	0.00	29	100.00	n/a	29	78.38
	**Normal (>P25) – 0 points in panel**	7	100.00	0	0.00		7	18.92
	**Not done**	0	0.00	0	0.00		1	2.70

**Handgrip**	**Low (<median 83.6)**	4	57.14	10	65.52	0.394	14	37.84
	**Strong (>=median 83.6)**	3	42.86	19	34.48		22	59.46
	**Not done**	0	0.00	0	0.00		1	2.70
**SC test**	**Slow (<median 217.95)**						Not done	
	**Quick (>=median 217.95)**							
	**Not well enough to perform it**							
**Breath test (n=19)**	**PEI (<=29)**							
	**Abnormal (>29)**							
	**Median result (range) (95 % CI)**							
**Faecal elastase**	**PEI (<200)-- 1 point in panel**	5	71.43	12	41.38	n/a	17	45.95
	**No PEI (>=200) – 0 point s in panel**	1	14.29	6	20.69		7	18.92
	**Not performed**	1	14.29	11	41.38		12	32.43
	**Median result (range) (95 % CI)**	15 (15-428) (0-260.78) 6 observations		50.5 (15-500) (71.04-262.40) 18 observations		0.3579	21.5 (15-500) (67.13-224.87) 24 observations	
**Patient treatments and outcomes**								
**Treatment intention**	**Palliative**	7	100.00	29	100.00	n/a	37	100.00
**Did patient receive systemic treatment**	**No**	1	14.29	9	31.03	0.645	11	29.73
	**Yes**	6	85.71	20	68.97		26	70.27
**Line of treatment**	**First-line**	6	85.71	19	65.52	1.000	25	67.57
	**Other line**	0	0.00	1	3.45		1	2.70
	**None**	1	14.29	9	31.03		11	29.73
**Type of systemic treatment**	**Gemcitabine**	1	14.29	6	20.69	n/a	7	18.92
	**FOLFIRINOX**	1	14.29	4	13.79		5	13.51
	**GemCap**	2	28.57	5	17.24		7	18.92
	**GemNabPaclitaxel**	2	28.57	3	10.34		5	13..51
	**Sunitinib**	0	0.00	0	0.00		0	0.00
	**SSA**	0	0.00	1	3.45		1	2.70
	**TemCap**	0	0.00	0	0.00		0	0.00
	**Carboplatin and Etoposide**	0	0.00	0	0.00		0	0.00
	**Other***	0	0.00	1	3.45		1	2.70
	**None**	1	14.29	9	31.03		11	29.73
***If Other (which)**	**NUC1031**	0	-	0	-		0	-
	**CisGem**	0	-	1	-		1	-
	**FOLFOX+NabPaclitaxelk**	0	-	0	-		0	-
**Chemotherapy dose intensity (%)**	**Median (range) (95% CI)**	56.45 (33.3-100) (36.59-84.68)		52.4 (11.1-77.8) (36.41-56.5)		0.1688	52.6 (11.1-100) (40.88-58.84)	
**Best radiological response**	**Progressive disease**	1	14.29	2	6.90	1.000	3	8.11
	**Stable disease**	4	54.17	8	27.59		12	32.43
	**Partial response**	1	14.29	3	10.34		4	10.81

	**Not documented or no treatment received**	1	14.29	16	55.17		18	48.65
**Radiological progression documented at time of last data lock**	**Yes**	1	14.29	4	13.79	1.000	5	13.51
**Death documented at time of last data lock**	**Yes**	1	14.29	13	44.83	0.209	15	40.54
**OS (estimated)**	**Median (95% CI)**	Nr (0.33-nr)		2.89 (1.93-nr)		0.2002	4.27 (2.17-nr)	
**Follow-up**	**Median (range) (95% CI)**	2.76 (0.33-3.42) (1.61-3.56)		2.33 (0-13.21) (1.57-3.49)		0.9565	2.49 (0-13.21) (1.75-3.27)	
**Nutritional blood panel**								
**Blood panel performed**	**No**	0	0.00	1	3.45	1.000	1	2.70
	**Yes**	7	100.00	28	96.55		36	97.30
**Ferritin**	**Normal**	-	-	-	-	-	-	-
	**Low**	-	-	-	-	-	-	-
	**High**	-	-	-	-	-	-	-
	**Not done**	-	-	-	-	-	-	-
**LDL**	**Normal**	-	-	-	-	-	-	-
	**High**	-	-	-	-	-	-	-
	**Not done**	-	-	-	-	-	-	-
**Vitamin D**	**Normal**	3	42.86	17	58.62	0.410	21	56.76
	**Low**	4	57.14	10	34.48		14	37.84
	**Not done**	0	0.00	2	6.90		2	5.41
**Total cholesterol**	**Normal**	-	-	-	-	-	-	-
	**High**	-	-	-	-	-	-	-
	**Not done**	-	-	-	-	-	-	-
**Triglycerides**	**Normal**	-	-	-	-	-	-	-
	**High**	-	-	-	-	-	-	-
	**Not done**	-	-	-	-	-	-	-
**HbA1c**	**Continuous variable median (range) (95% CI)**	41 (28-64) (19.11-67.89)		40 (25-133) (32.74-60.86)		0.8093	40 (25-133) (34.49-56.01)	
**Vitamin E**	**Normal**	7	100.00	26	89.66	n/a	34	91.89
	**Low**	0	0.00	0	0.00		0	0.00
	**Not done**	0	0.00	3	10.34		3	8.11
**Vitamin A**	**Normal**	6	85.71	17	58.62	0.397	23	62.16
	**Low**	1	14.29	9	31.03		11	29.73
	**Not done**	0	0.00	3	10.34		3	8.11
**Amylase**	**Normal**							
	**Low**							
	**High**							
	**Not done**							
**Magnesium**	**Normal**	6	85.71	26	89.66	0.374	32	86.49
	**Low**	1	14.29	1	3.45		3	8.11
	**Not done**	0	0.00	2	6.90		2	5.41
**Transferrin**	**Normal**	-	-	-	-	-	-	-
	**Low**	-	-	-	-	-	-	-
	**Not done**	-	-	-	-	-	-	-
**INR**	**Normal**	3	42.86	6	20.69	0.295	9	24.32
	**High**	2	28.57	16	55.17		19	51.35
	**Not done**	2	28.57	7	24.14		9	24.32

**Hb**	**Normal**	6	85.71	21	72.41	1.000	27	72.97
	**Low**	1	14.29	7	24.14		9	24.32
	**Not done**	0	0.00	1	3.45		1	2.70
**MCV**	**Normal**	5	71.43	23	79.31	0.608	28	75.68
	**Low**	0	0.00	0	0.00		0	0.00
	**High**	2	28.57	5	17.24		8	21.62
	**Not done**	0	0.00	1	3.45		1	2.70
**Serum total protein**	**Normal**	7	100.00	25	86.21	1.000	33	89.19
	**Low**	0	0.00	3	10.34		3	8.11
	**Not done**	0	0.00	1	3.45		1	2.70
**Albumin**	**Normal**	6	85.71	27	93.10	0.365	33	89.19
	**Low**	1	14.29	1	3.45		3	8.11
	**Not done**	0	0.00	1	3.45		1	2.70
**Lymphocytes**	**Normal (>=1500)**	3	42.86	15		0.691	18	48.65
	**Low**	4	57.14	13	451.724. 83		18	48.65
	**Not done**	0	0.00	1	3.45		0	0.00
**Neutrophils**	**Normal (2000-7500)**	6	85.71	20	68.97	0.648	27	72.97
	**Low**	0	0.00	0	0.00		0	0.00
	**High**	1	14.29	8	27.59		9	24.32
	**Not done**	0	0.00	1	3.45		1	2.70
**NLR**	**Low (below median; <4.25)**	4	57.14	13	44.83	0.684	17	45.95
	**High (above median; >=4.25)**	3	42.86	16	55.17		20	54.05
	**Not done**	0	0.00	0	0 00		0	0.00

10. Supplementary material S10: Changes over time (follow-up cohort).

All percentages calculated at per ITT. Missing data as a category to try and adjust for selection bias

Abbreviations: BMI, body mass index; ECOG-PS, Eastern Cooperative Oncology Group Performance Status; FAACT A/CS, functional assessment of anorexia/cachexia therapy anorexia/cachexia subscale; MUAC, mid-upper arm circumference; NET, neuroendocrine tumour; PEI, pancreatic exocrine insufficiency; PERT, pancreatic enzyme replacement therapy; PPI, proton pump inhibitor; VAS, visual analogue scale.

Grade defined as the severity of the adverse event as per CTCAE v5.0. Grade 1 – mild symptoms or asymptomatic with no interventional indicated. Grade 2 – moderate symptoms, limiting age-appropriate self-care and minimal intervention required. Grade 3 – severe symptoms but not immediately life-threatening, limiting self-care and hospitalisation required.

		**Baseline visit (n=37)**		**Week 6 visit (n=37)**		**Month 3 visit (n=37)**	
		**n**	**%**	**n**	**%**	**n**	**%**
**Visit completed**	**yes**	37	100.00	20	54.05	17	45.94
		**Patient characteristics**					
**ECOG -PS at study entry**	**0**	6	16.22	1	2.70	0	0.00
	**1**	21	56.76	15	40.54	14	37.84
	**2**	7	18.92	3	8.11	2	5.41
	**3**	3	8.11	0	0.00	0	0.00
	**4**	0	0.00	0	0.00	0	0.00
	**Not assessed**	0	0.00	18	48.65	21	56.76

**PEI and PERT**							
**PEI present at study entry**	**No**	4	10.81	3	8.11	4	10.81
	**Yes**	26	70.27	15	40.54	13	35.14
	**Not assessed**	7	18.92	19	51.35	20	54.05
**PEI grade**	**Grade 1**	13	35.14	11	-	13	-
	**Grade 2**	13	35.14	3	-	0	-
	**Grade 3**	0	0.00	0	-	0	-
	**Not assessed**	11	29.73	23	-	24	-
**PEI diagnosis based on**	**Symptoms**	1	2.70	1	2.70	2	5.41
	**Symptoms + risk panel**	25	67.57	14	37.84	13	29.73
	**No PEI/not assessed**	(4/7) 11	29.73	(3/19) 22	59.46	24	64.86
**Is PEI diagnosed since study entry?**	**Yes**	n/a	-	1	2.70	0	0.00
	**No**	n/a	-	16	43.24	16	43.24
	**n/a**	n/a	-	20	54.05	21	56.76
**PERT at time of or following visit**	**No**	5	13.51	4	10.81	1	2.70
	**Yes**	25	67.57	14	37.84	16	43.24
	**Not assessed**	7	18.92	19	51.35	20	54.05
**PERT started by dietitian**	**No**	22	59.46	17	45.95	17	45.95
	**Yes**	6	16.22	1	2.70	0	0.00
	**Not documented**	9	24.32	19	51.35	20	54.05
**PERT dose adjusted by dietitian at study entry**	**No**	17	45.95	14	37.84	14	37.84
	**Yes**	10	27.03	4	10.81	3	8.11 (input required to modify dose rather than start, they should be on it already)
	**Not applicable**	10	27.03	19	51.35	20	54.05
**PERT-related toxicity**	**No**	n/a	-	12	32.43	16	43.24
	**Yes**	n/a	-	1 (itchy skin)	2.70; 1/13; 7.69% (per protocol)	0	0.00
**PERT compliance**	**50**	n/a	-	1	2.70		
	**75**	n/a	-	0	0.00	1	2.70
	**90**	n/a	-	1	2.70	1	2.70
	**100**	n/a	-	9	24.32	5	13.51
	**Not assessed**	n/a	-	26	70.27	30	81.08
**Patient on PPI**	**No**	13	35.14	5	13.51	3	8.11
	**Yes**	17	45.95	12	32.43	14	37.84
	**Not assessed**	7	18.92	20	54.05	20	54.05
**Symptoms**							
**PEI symptoms assessed (dietitian assessment)**	**Yes**	30	81.08	18	48.65	17	45.95
	**No**	7	18.92	19	51.36	20	54.05
	**Yes**	26	70.27	15	40.54	12	32.43

**Symptoms in keeping with PEI**	**No**	4	10.81	3	8.11	5	13.51
	**Not assessed**	7	18.92	19	51.35	20	54.05
**Steatorrhea**	**Yes**	16	43.24	9	24.32	7	18.92
	**Grade 1**	10	-	7	-	6	-
	**Grade 2**	5	-	1	-	0	-
	**Grade 3**	0	-	0	-	0	-
	**No**	21	56.76	11	29.73	10	27.03
**Weight loss**	**Yes**	27	72.97	11	29.73	10	27.03
	**No**	10	27.03	9	24.32	7	18.92
**Absolute amount of weight loss (kg)**	**Median (range) (95% CI)**	10.75 (1-35.5) (8.33-14.75)		2 (0.4-7.5) (1.51-5.09)		1.95 (0.5-5.75) (1.32-4.32)	
**Relative amount of weight loss (%)**	**Median (range) (95% CI)**	13 (1.4-37.2) (10.63-18.03)		3.2 (0.4-12.2) (2.13-7.57)		2.95 (0.94-8.7) (1.99-6.38)	
**Time period of weight loss (months)**	**Median (range) (95% CI)**	3.5 (1-12) (3.38-6.04)		1.5 (1-6) (0.92-2.80)		1.5 (1-1.75) (1.29-1.68)	
**Flatus indigestion**	**Yes**	26	70.27	16	43.24	12	32.43
	**Grade 1**	18	-	12	-	9	-
	**Grade 2**	2	-	1	-	0	-
	**Grade 3**	1	-	0	-	0	-
	**No**	11	29.73	4	10.81	5	13.51
**Abdominal discomfort**	**Yes**	19	51.35	5	13.51	4	10.81
	**Grade 1**	12	-	5	-	4	-
	**Grade 2**	2	-	0	-	0	-
	**Grade 3**	1	-	0	-	0	-
	**No**	18	48.65	15	40.54	13	35.14
**Bowel movements at study entry**	**Frequency <1/day**	6	16.22	0	0.00	0	0.00
	**Frequency 1/day**	9	24.32	14	37.84	11	29.73
	**Frequency 2-3/day**	10	27.03	0	0.00	3	8.11
	**Frequency >3/day**	3	8.11	1	2.70	1	2.70
**Bowel movements at study entry**	**Type 1**	4	10.81	2	5.41	1	2.70
	**Type 2**	2	5.41	2	5.41	3	8.11
	**Type 3**	3	8.11	2	5.41	0	0.00
	**Type 4**	12	32.43	8	21.62	8	21.62
	**Type 5**	0	0.00	0	0.00	3	8.11
	**Type 6**	6	16.22	3	8.11	2	5.41
	**Type 7**	3	8.11	2	5.41	0	0.00
	**Not assessed**	7	18.92	18	48.65	20	54.05
**Bowel movements at study entry**	**Type 1-5**	21	56.76	14	37.84	15	40.54
	**Type 6-7**	9	24.32	5	13.51	2	5.41
**Other**	**Pain**	1	-	0	-	0	-
	**Early satiety**	0	-	0	-	0	-
**Dietitian related assessment**							
**Weight (kg)**	**Median (range) (95% CI)**	66.1 (44.1-100.9) (62.99-71.48)		66.70 (52.4-99.6) (62.04-74.92)		60.25 (52.3-109.9) (59.10-75.35)	
**BMI**	**Median (range) (95% CI)**	23.43 (16.70-33.75) (22.46-25.29)		24.55 (18.35-31.19) (22.75-25.96)		22.80 (19.01-34.42) (22.22-26.23)	
	**Low (<18.5)**	3	8.11	1	2.70	0	0.00
**FAACT questionnaire completed**	**Yes**	34	91.89	18	48.65	13	35.14
**Low appetite**	**Yes (based on points +/- vas)**	29	78.38	12	32.43	12	32.43

**(based on FAACT-A/C-S)**	**Yes; FAACT <=37 points**	26	70.27	11	29.73	11	29.73
	**No ; FAACT >37 points**	8	21.62	7	18.92	1	2.70
	**Yes; VAS <=7 cm**	21	56.76	11	29.73	8	21.62
	**No ; VAS>7 cm**	11	29.73	6	16.22	3	8.11
**MUAC**	**Low (<=P25) – 2 points in panel**	29	78.38	16	43.24	12	32.43
	**Normal (>P25) – 0 points in panel**	7	18.92	3	8.11	4	10.81
	**Not done**	1	2.70	18	48.65	21	56.76
**Handgrip**	**Low (<median 83.6)**	14	37.84	9	24.32	11	29.73
	**Strong (>=median 83.6)**	22	59.46	10	27.03	5	13.51
	**Not done**	1	2.70	18	48.65	21	56.76
**Faecal elastase**	**PEI (<200)-- 1 point in panel**	17	45.95	12	32.43	4	10.81
	**No PEI (>=200) – 0 points in panel**	7	18.92	3	8.11	4 (jncluding 3 missing)	10.81
	**Not performed**	13	35.14	22	59.46	29	78.38
	**Median result (range) (95 % CI)**	21.5 (15-500) (67.13-224.87) (24 observations)		22 (15-466) (27.08-200.39) (15 observations)		189 (48-444) (14.01-384.79) 5 observations	
**Screening panel**							
**Panel fully completed (MUAC and FE) (n=37)**	**No**	13 (13 missing FE, 1 missing FE-1 and MUAC)	35.14	5	13.51	12	32.43
	**Yes**	24	64.86	15 (5/20 75.00%; at this point still; feasible to repeat it	40.54	5	13.51; 5/17 (29.4%); not very feasible to repeat over time, one assessment enough
**Screening panel results (if missing values this could be calculated anyway)**	**0 points**	2	5.41	1	2.70	3	8.11
	**1 point**	5	13.51	2	5.41	1	2.70
	**2 points**	17	45.95	6	16.22	9	24.32
	**3 points**	12	32.43	10	27.03	3	8.11
	**Not assessed**	1	2.70	18	48.65	21	56.76
**Panel result (if missing values this could be calculated anyway)**	**Low risk (0/1 points)**	7	18.92	3	8.11	4	10.81
	**High risk (2/3 points)**	29	78.38	16	43.24	12	32.43
	**Not assessed**	1	2.70	18	48.65	21	56.76
**Agreement dietitian and panel**	**No**	3/29* *disagreement reason: low MUAC due to poor nutritional intake secondary	10.34	3*/17 *1 due to presence of clear steatorrhoea, 1 pts difficulties to assess symptoms	17.65	4*/16 *1 not specified, 1 pts low MUAC due to poor nutrition; 2 pts low panels due to missing FE	25.00

		to pain and stress (n=1), no symptoms and normal FE-1 despite high-risk panel (n=1), symptoms difficult to assess due newly formed ileostomy (n=1)		due to ileostomy, FE-1 normal, 1 pts			
	**Yes**	26/29	89.66	14/17	82.35	12/16	75.00
	**N/a (7 did not have dietitian assessment and in 1 pts panel could not be calculated so these are excluded)**	8	-	20	-	21	-
			**Blood test**				
**Blood panel performed**	**No**	1	2.70	18	48.65	20	54.05
	**Yes**	36	97.30	19	51.35	17	45.95
**HbA1c**	**Continuous variable median (range) (95% CI)**	40 (25-133) (34.49-56.01)		-		-	
**Vitamin D**	**Normal**	21	56.76	14	37.84	11	29.73
	**Low**	14	37.84	4	10.81	2	5.41
	**Not done**	2	5.41	19	51.35	24	64.86
**Vitamin E**	**Normal**	34	91.89	16	43.24	10	27.03
	**Low**	0	0.00	0	0.00	0	0.00
	**Not done**	3	8.11	21	56.76	27	72.97
**Vitamin A**	**Normal**	23	62.16	12	32.43	9	24.32
	**Low**	11	29.73	4	10.81	1	2.70
	**Not done**	3	8.11	21	56.76	27	72.97
**Magnesium**	**Normal**	32	86.49	15	40.54	14	37.84
	**Low**	3	8.11	3	8.11	1	2.70
	**Not done**	2	5.41	19	51.35	22	59.46
**INR**	**Normal**	9	24.32	0	0.00	0	0.00
	**High**	19	51.35	1	2.70	0	0.00
	**Not done**	9	24.32	36	97.30	37	100.00
**Hb**	**Normal**	27	72.97	7	18.92	3	8.11
	**Low**	9	24.32	12	32.43	14	37.84
	**Not done**	1	2.70	18	48.65	20	54.05
**MCV**	**Normal**	28	75.68	18	48.65	13	35.14
	**Low**	0	0.00	0	0.00	0	0.00
	**High**	8	21.62	1	2.70	4	10.81
	**Not done**	1	2.70	18	48.65	20	54.05
**Serum total protein**	**Normal**	33	89.19	14	37.84	14	37.84
	**Low**	3	8.11	5	13.51	3	8.11
	**Not done**	1	2.70	18	48.65	20	54.05
**Albumin**	**Normal**	33	89.19	19	51.35	16	43.24
	**Low**	3	8.11	0	0.00	1	2.70
	**Not done**	1	2.70	18	48.65	20	54.05
**Lymphocytes**	**Normal (>=1500)**	18	48.65	10	27.03	8	21.62
	**Low**	18	48.65	9	24.32	9	24.32
	**Not done**			18	48.65	20	54.05
**Neutrophils**	**Normal (2000-7500)**	27	72.97	14	37.84	16	43.24

	**Low**	0	0.00	3	8.11	0	0.00
	**High**	9	24.32	2	5.41	1	2.70
	**Not done**	1	2.70	18	48.65	20	54.05
**NLR**	**Low (below median; <4.25)**	17	45.95	15	40.54	12	32.43
	**High (above median; >=4.25)**	20	54.05	5	13.51	5	13.51
	**Not done**	0	0.00	17	45.95	20	54.05

11. Supplementary material S11: Changes in the screening panel over time

Changes of screening panel results between baseline (left side), week 6 (middle) and month 3 (right side) are provided.

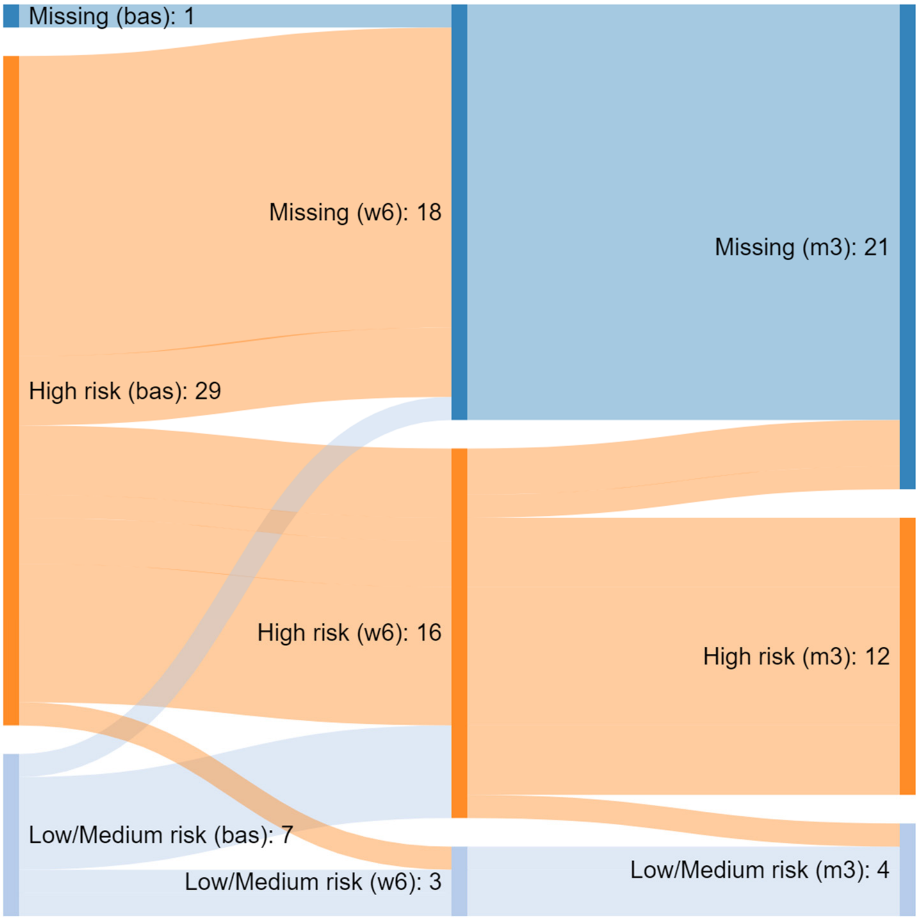

12. Supplementary Material S12: 12.A: Quality of life data at baseline and changes over time. 12.B.: Symptom scale data at baseline and changes over time (patients with pancreatic ductal adenocarcinoma are shown)

12. A

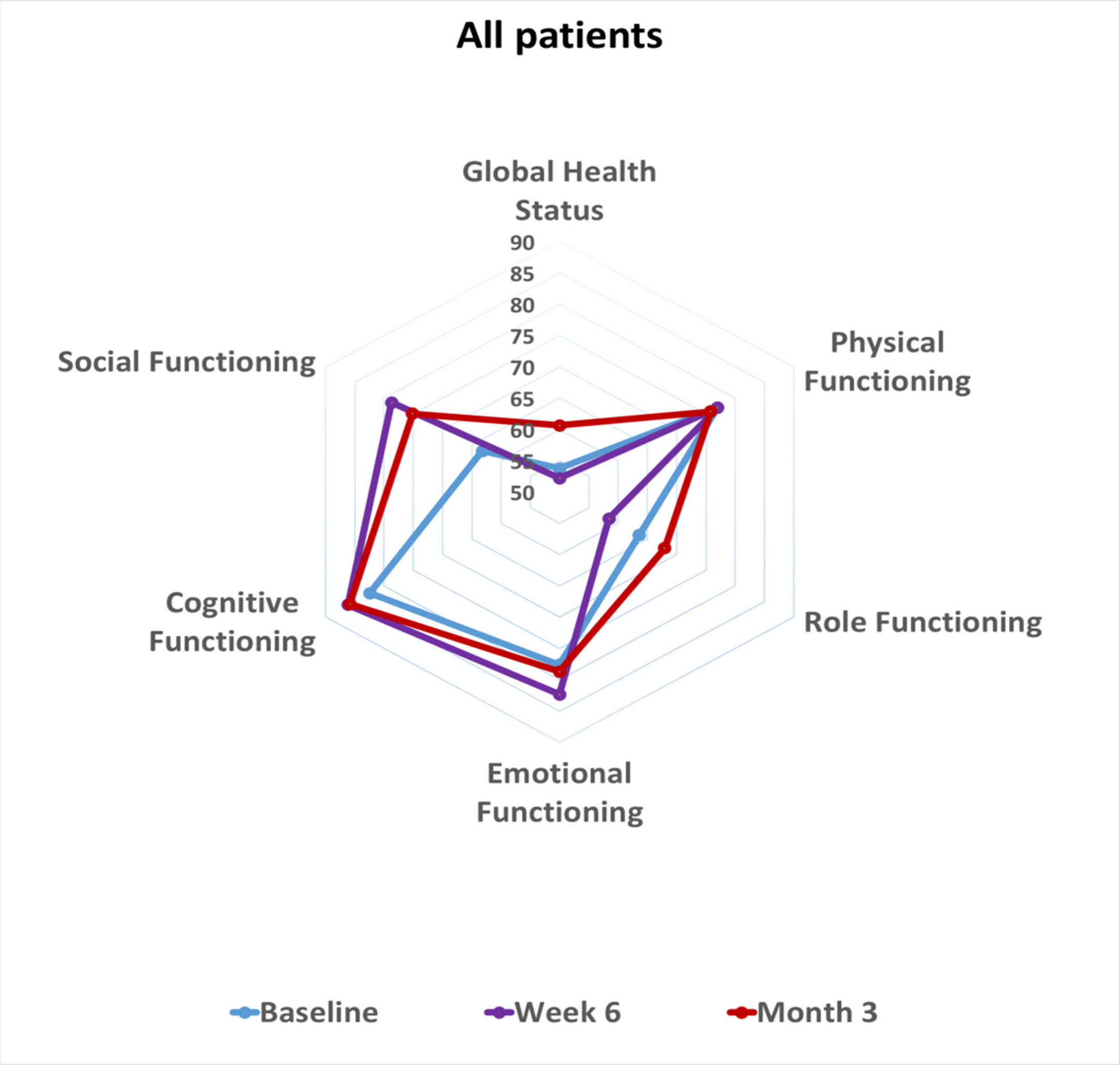
All patients
Global Health Status
90
85
80
75
70
65
60
55
50
Physical Functioning
Role Functioning
Emotional Functioning
Cognitive Functioning
Social Functioning
Baseline
Week 6
Month 3

12. B

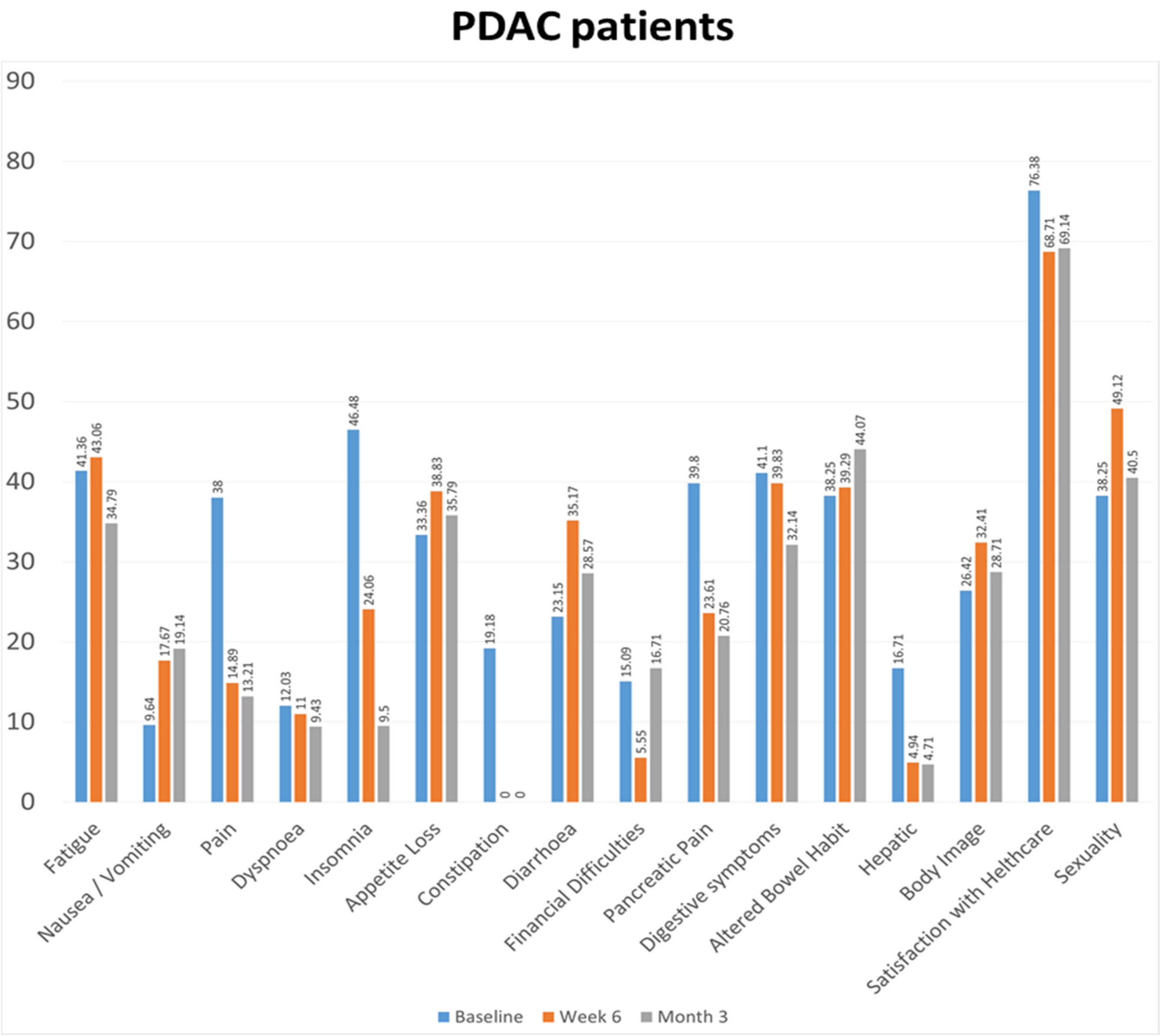
PDAC patients
90
80
70
60
50
40
30
20
10
0
Fatigue 41.36 43.06 34.79
Nausea / Vomiting 9.64 17.67 19.14
Pain 38 14.89 13.21
Dyspnoea 12.03 11 9.43
Insomnia 46.48 24.06 9.5
Appetite Loss 33.36 38.83 35.79
Constipation 19.18 0 0
Diarrhoea 23.15 35.17 28.57
Financial Difficulties 15.09 5.55 16.71
Pancreatic Pain 39.8 23.61 20.76
Digestive symptoms 41.1 39.83 32.14
Altered Bowel Habit 38.25 39.29 44.07
Hepatic 16.71 4.94 4.71
Body Image 26.42 32.41 28.71
Satisfaction with Helthcare 76.38 68.71 69.14
Sexuality 38.25 49.12 40.5
Baseline
Week 6
Month 3
